# Reactive Oxygen Species Modulation of Na/K-ATPase Regulates Fibrosis and Renal Proximal Tubular Sodium Handling

**DOI:** 10.1155/2012/381320

**Published:** 2012-02-23

**Authors:** Jiang Liu, David J. Kennedy, Yanling Yan, Joseph I. Shapiro

**Affiliations:** ^1^Department of Medicine, College of Medicine University of Toledo, 3000 Arlington Avenue, Toledo, OH 43614, USA; ^2^Department of Cell Biology, Lerner Research Institute, Cleveland Clinic, 9500 Euclid Avenue, Cleveland, OH 44195, USA; ^3^Institute of Biomedical Engineering, Yanshan University, Qinhuangdao 066004, China

## Abstract

The Na/K-ATPase is the primary force regulating renal sodium handling and plays a key role in both ion homeostasis and blood pressure regulation. Recently, cardiotonic steroids (CTS)-mediated Na/K-ATPase signaling has been shown to regulate fibrosis, renal proximal tubule (RPT) sodium reabsorption, and experimental Dahl salt-sensitive hypertension in response to a high-salt diet. Reactive oxygen species (ROS) are an important modulator of nephron ion transport. As there is limited knowledge regarding the role of ROS-mediated fibrosis and RPT sodium reabsorption through the Na/K-ATPase, the focus of this review is to examine the possible role of ROS in the regulation of Na/K-ATPase activity, its signaling, fibrosis, and RPT sodium reabsorption.

## 1. Introduction

According to the American Heart Association (AHA), over 70 million people in the United States aged 20 and older have high blood pressure (BP). The cause of 90–95 percent of the cases of high BP is unknown, yet in the last decade the associated morbidity and mortality from high BP has increased precipitously. In the 2008 AHA Scientific Statement [1], excessive dietary salt intake is listed as one of the major lifestyle factors which significantly contributes to the development of hypertension and tends to be more pronounced in typical salt-sensitive patients. Modest dietary salt restriction and diuretic therapy, therefore, are recommended for treatment of resistant hypertension, especially in the salt-sensitive subgroup [1, 2]. Renal sodium handling is a key determinant of long-term BP regulation [3]. The relationship between dietary sodium, salt sensitivity, and BP has been established on an epidemiological and clinical basis. It is estimated that hypertension affects 25% to 35% of the world population aged 18 and older [4], and more hypertensive subjects (~50%) are significantly salt sensitive than normotensive subjects (~25%) [5]. In the DASH-Sodium clinical trial, BP reduction was correlated with sodium restriction in the salt-sensitive subjects regardless of diet [6]. Interestingly, animal renal cross-transplantation experiments [7–10] and studies of human renal transplantation [11] demonstrate that BP levels “travel with the donor's kidney,” providing compelling evidence for the role of renal function in the pathogenesis of hypertension. In clinical and experimental models, renal proximal tubule (RPT) sodium handling accounts for over 60% reabsorption of filtered sodium and is an independent determinant of BP response to salt intake, playing a critical role in the pathogenesis of salt-sensitive hypertension. Recently, accumulating data indicate that cardiotonic steroids (CTS) signaling through the Na/K-ATPase contribute to RPT sodium handling and salt sensitivity [12–18].

## 2. CTS and Na/K-ATPase Signaling

CTS (also known as endogenous digitalis-like substances) include plant-derived digitalis drugs, such as digoxin and ouabain, and vertebrate-derived aglycones such as bufalin and marinobufagenin (MBG) [16, 18]. Recent studies have identified both ouabain and MBG as endogenous steroids whose production, and secretion are regulated by stimuli including angiotensin II (Ang II) [18–21]. The Na/K-ATPase belongs to the P-type ATPases family and consists of two noncovalently linked *α* and *β* subunits [22–24]. Several *α* and *β* isoforms, expressed in a tissue-specific manner, have been identified and functionally characterized [22–25]. The *α*1 subunit contains multiple structural motifs that interact with soluble, membrane and structural proteins including Src, caveolin-1, phospholipase C-*γ*, PI3 kinase, IP3 receptor, BiP, calnexin, cofilin, and ankyrin [26–36]. Binding to these proteins not only regulates the ion-pumping function of the enzyme, but it also conveys signal-transducing functions to the Na/K-ATPase [18, 32, 37–39]. In LLC-PK1 cells, the Na/K-ATPase *α*1 subunit and Src form a functional receptor in which the binding of CTS to the *α*1 subunit activates Src and consequent signaling cascade [39]. The signaling function of the Na/K-ATPase regulates numerous cell functions in various types of organs and cells including cell motility, cell proliferation, cancer, endothelin release, glycogen synthesis, apoptosis, hypertension, intracellular calcium signaling, cardiac hypertrophy, cardiac remodeling, renal remodeling, epithelial cell tight junction, vascular tone homeostasis, and sodium homeostasis [26, 40–59]. The topic of CTS-Na/K-ATPase signaling and its downstream pathophysiological implications has been extensively reviewed in the last few years [12–16, 18, 21, 32, 39, 59–64], and we will not discuss them in detail in this review.

## 3. CTS and Sodium Homeostasis

Endogenous CTS were first proposed many years ago to function as a natriuretic hormone. Although their pathophysiological significance has been a subject of debate for many years [65], the concept of a natriuretic hormone is supported by the experimental observations in animal models of ouabain-induced natriuresis [66, 67]. Recently, several elegant reports have confirmed this concept with different approaches. Gene replacement studies have unequivocally demonstrated an important role of endogenous CTS in regulation of renal sodium excretion and BP [68, 69]. In transgenic mice expressing ouabain-sensitive Na/K-ATPase ***α*** 1 subunit, a significant observation is an augmented natriuretic response to both acute salt load and ouabain infusion, indicating that the ouabain-binding site of Na/K-ATPase ***α*** 1 subunit participates in the natriuretic response to salt load by responding to endogenous ouabain. Moreover, the augmented natriuretic response in the ouabain-sensitive ***α*** 1 isoform mice can be blocked with administration of an anti-digoxin antibody fragment [68, 69]. In normal male Wistar rats, endogenous circulatory ouabain has physiological roles controlling vasculature tone and sodium homeostasis, showing endogenous ouabain regulates renal sodium excretion in normal animals [70]. A significant inhibition of natriuresis is observed in rats that were passively immunized with anti-ouabain antibody and in rats that were actively immunized with ouabain-albumin antigen, in which endogenous ouabain levels were reduced in both cases. Like ouabain, MBG has both natriuretic and vasoconstrictor effects [12, 71, 72]. High salt intake induced an initial transient stimulation of ouabain and a subsequent progressive increase of MBG both in Dahl salt-sensitive (S) rats and in humans [12, 73]. In Dahl S rats, the increase in natriuresis stimulated by an acute salt loading is prevented by administration of both an anti-ouabain antibody and an anti-MBG antibody [72]. Furthermore, endogenous CTS have also been implied in age-related increases in salt sensitivity of BP in human normotensive subjects [73]. It is estimated that approximately 50% of humans with untreated essential hypertension have significantly elevated levels of endogenous ouabain [74] which is involved in the regulation of vascular tone homeostasis through stimulation of interaction between the Na/K-ATPase and sodium/calcium exchanger [59]. In normotensive human males, both a high salt diet and systematic sodium depletion (by hydrochlorothiazide) significantly increase plasma ouabain concentration [75].

Release of endogenous ouabain from the brain hypothalamus stimulated by a high salt diet leads to inhibition of the Na/K-ATPase activity and central sympathetic activation (reviewed in [76, 77]), which plays a crucial role in the pressor effects of high salt intake in spontaneously hypertensive rats (SHR) and Dahl S rats. In Dahl S rats, elevation in brain ouabain increased MBG secretion from the adrenal cortex, and this effect was blocked by the AT1 receptor antagonist losartan [78, 79]. The data suggest that the observed CTS-induced natriuretic effect might be a result of the combined effects of ouabain and MBG [60]. Furthermore, CTS not only induced hypertension in rats but also caused significant cardiovascular remodeling and natriuresis independent of their effect on BP [49, 51, 70, 80, 81].

## 4. CTS and Oxidative Stress in Cardiac and Renal Fibrosis

**Figure 1:**
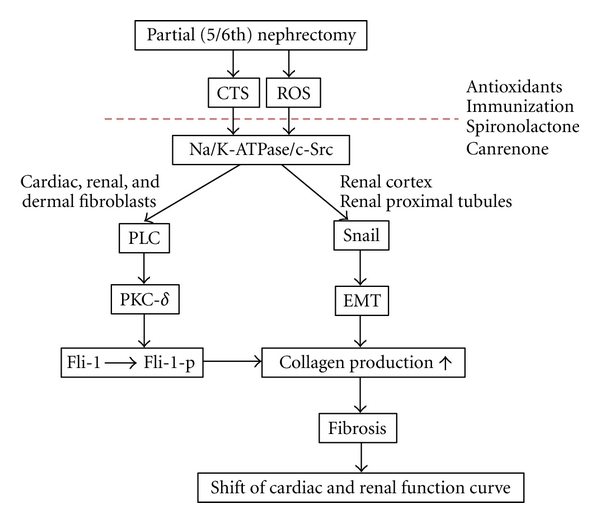
Schematic illustration of the effect of CTS on fibrosis.

CTS binding to the Na/K-ATPase induces cellular reactive oxygen species (ROS) production and its downstream effects, such as cardiac and renal fibrosis, and these effects can be prevented by ROS scavenging [80, 82–84]. In kidney and heart, the central role of CTS in the development of fibrosis has been demonstrated both *in vivo*, in the partial (5/6th) nephrectomy model, and *in vitro* cell culture, including cardiac and renal fibroblasts. 5/6th nephrectomy increases circulating levels of MBG and stimulates cardiac fibrosis in both rat and mouse [80, 84, 85]. Rats subjected to 5/6th nephrectomy develop systemic oxidant stress that is similar to that seen in rats subjected to MBG infusion as evidenced by significant elevation of plasma carbonylated protein. Active immunization against MBG and reduction of circulating levels of MBG by adrenalectomy substantially attenuate 5/6th nephrectomy and MBG infusion-mediated cardiac fibrosis and oxidant stress, an effect that is independent of BP [80, 84]. In primary culture of rat cardiac and human dermal fibroblasts as well as a cell line derived from rat renal fibroblasts, MBG and ouabain stimulate [^3^H]proline incorporation as well as gene and protein expression of collagen [86]. MBG induced a PLC-dependant translocation of PKC-*δ* to the nucleus, resulting in the phosphorylation and degradation of transcription factor Friend leukemia integration-1 (Fli-1), a negative regulator of collagen synthesis [86]. Both CTS-induced Na/K-ATPase signaling and oxidative stress are necessary in the pathogenesis of cardiac and renal fibrosis as evidenced by CTS-stimulated phosphorylation of both Src and MAPK which is effectively blocked not only by ROS scavenging and Src inhibition but also through possible competitive inhibition of CTS binding to Na/K-ATPase by spironolactone and canrenone [80, 84, 87, 88]. MBG infusion causes renal fibrosis mainly in the cortex of the kidney by stimulation of the transcription factor Snail expression and its nuclear localization in the tubular epithelia, which is associated with epithelial-to-mesenchymal transition (EMT) during renal fibrosis [89]. This EMT phenomenon is also demonstrated in LLC-PK1 cells, indicating that MBG may cause damage of renal proximal tubules. CTS-induced fibrosis in heart and kidney might shift the cardiac and renal function curve to favor a higher set point of pressure natriuresis (Figure 1).

## 5. Na/K-ATPase Signaling and RPT Sodium Handling

It has been postulated for decades that endogenous CTS stimulated by increased sodium intake increases natriuresis and diuresis by directly inhibiting renal tubular Na/K-ATPase to prevent renal reabsorption of filtered sodium [90–92]. There is accumulating evidence supporting this idea under conditions such as high salt intake, chronic renal sodium retention, renal ischemia, uremic cardiomyopathy, and volume expansion in various animal models and human beings [12, 13, 15, 60, 62, 75, 93–107]. Although the direct inhibition of the Na/K-ATPase enzymatic activity and sodium reabsorption in RPTs by CTS has not been validated, recent observations indicate that ligand-mediated RPT sodium reabsorption via Na/K-ATPase/c-Src signaling counterbalances the sodium retention-mediated increases in BP, such as that seen in salt-sensitive hypertension [108–114]. Ouabain, a ligand of the Na/K-ATPase, activates the Na/K-ATPase/c-Src signaling pathway and subsequently redistributes basolateral Na/K-ATPase and apical sodium/hydrogen exchanger isoform 3 (NHE3) in RPTs, leading to reduced RPT sodium reabsorption and increased urinary sodium excretion.

In LLC-PK1 cells, ouabain activates Na/K-ATPase/c-Src signaling pathways and reduces cell surface Na/K-ATPase and NHE3, leading to a significant inhibition of active transcellular ^22^Na^+^ flux from the apical to basolateral compartment [108–112]. MBG, an important CTS species, and deproteinated extract of serum (derived from patients with chronic renal failure) also induce Na/K-ATPase endocytosis and inhibition of active transepithelial ^22^Na^+^ flux. However, it is still not clear how ouabain-activated Na/K-ATPase signaling regulates NHE3, since, at concentrations of ouabain used, no significant change in intracellular Na^+^ concentration was observed [108]. Interestingly, this phenomenon is either not observed or is much less significant in MDCK cells (a canine renal distal tubule cell line). These *in vitro* data suggest that CTS-Na/K-ATPase signaling has a profound effect on RPT sodium handling, but not in distal tubules.

**Figure 2:**
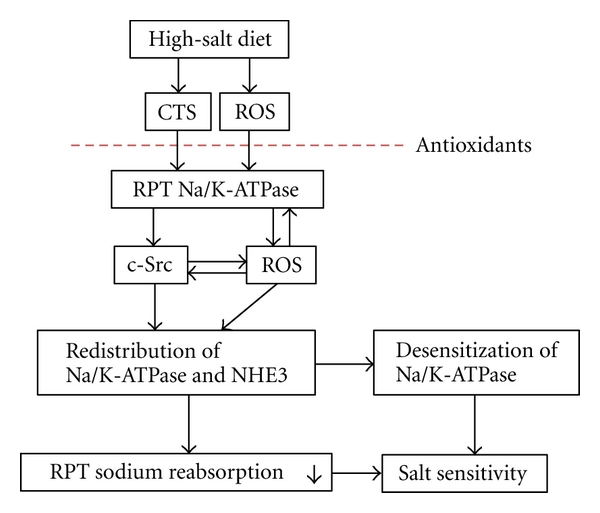
Schematic illustration of the effects of ROS and Na/K-ATPase signaling on RPT sodium reabsorption. CTS: cardiotonic steroids; RPT: renal proximal tubules.

Different species of endogenous CTS show differences in the kinetics and tissue actions in response to salt loading in both animal models and in human salt-sensitive hypertensive patients [16, 18, 60, 75, 79, 105]. The Dahl salt-resistant (R) and salt-sensitive (S) strains were developed by selective breeding of the outbred Sprague-Dawley rat strain for resistance or susceptibility to the hypertensive effects of high dietary sodium [115]. RPT sodium handling is a critical determinant of the different BP responses in these strains [7, 116–118], and there is no Na/K-ATPase *α*1 gene (*Atp1a1*) difference between R and S rats [119]. In comparison to Dahl S rats that eliminate excessive sodium mainly through pressure natriuresis at the expense of an elevated systolic BP, a major response to salt loading in the Dahl R rats is a greater reduction in renal sodium reabsorption to eliminate excessive sodium without raising BP. Our recent *in vivo* observation [114] is in agreement with this hypothesis and our *in vitro* observations in LLC-PK1 cells. Specifically in isolated RPTs, both a high salt diet and ouabain are able to activate Na/K-ATPase/c-Src signaling pathways, leading to the redistribution of Na/K-ATPase and NHE3 in the Dahl R but not in the S rats. The R rats show significant increases in total urinary sodium excretion and RPT-mediated fractional sodium excretion, without BP elevation [114]. While the BP response to salt loading in R and S rats involves many regulatory factors [117], our data indicate that impairment of the RPT Na/K-ATPase/c-Src signaling contributes to salt sensitivity. Since it failed to confirm the possible difference of Na/K-ATPase *α*1 gene (*Atp1a1*) between R and S rats [119], other factor(s) must be present to prevent activation of Na/K-ATPase/c-Src signaling in the S rats. The possible effect of CTS on RPT Na/K-ATPase signaling and sodium reabsorption is summarized in Figure 2.

 The SHR rat is an established model of human essential hypertension with the characteristic of vascular resistance. SHR rat develops hypertension spontaneously at the age of 7–15 weeks, regardless of salt loading, mainly through increase in peripheral vascular resistance [120] including renal vascular resistance. Interestingly, either reduction of dietary vitamin E or caloric restriction without sodium restriction prevents the development of hypertension [121, 122]. Like Dahl S rats, SHR rats eliminate excessive sodium via a pressure-natriuresis mechanism but have significant lower BP response to a high salt diet and higher systolic BP to eliminate the same amount of sodium when compared to Dahl S rats [123–126]. Most interestingly, comparing to control Wistar-Kyoto (WKY) rats, regulation of the Na/K-ATPase and NHE3 with both aging and oxidative stress have been shown contributed to the development and maintenance of hypertension in the SHR [127–135]. In renal cortical (proximal) tubules, activity and protein levels of NHE-3 are significantly higher in SHR than age-matched WKY rats at all stages during the development and maintenance of hypertension. When NHE3 function is determined by the rate of bicarbonate reabsorption by *in vivo* stationary microperfusion in RPT, young SHR rats show higher NHE3 activity than adult SHR and WKY rats, and this is accompanied by changes in NHE3 phosphorylation and distribution. In young SHR rats, the RPT Na/K-ATPase activity is significantly higher than in age-matched WKY, which can be prevented by treatment of the diuretic drug hydrochlorothiazide. However, the Na-K-ATPase activity in medullary thick ascending limb, cortical thick ascending limb, and distal tubule is significantly lower in young SHR rats than in age-matched WKY rats. There were no significant differences in Na/K-ATPase activity in these nephron segments in adult SHR and WKY rats, nor in collecting duct segments of young and adult SHR and age-matched WKY rats. Interestingly, however, the Na/K-ATPase ***α*** 1 subunit gene expression is lower in both young and adult SHR rats than age-matched WKY rats, indicating a posttranscriptional regulation.

 Recent study indicates that SHR rats have enhanced renal superoxide generation and NADPH oxidase (NOX) expression in both vascular and renal tissue before and after development of hypertension [136]. Elevated basal level of superoxide inhibits proximal tubule NHE3 activity and fluid reabsorption in SHR rats in comparison to WKY rats. This effect is prevented by the NOX inhibitor apocynin or knockdown of the critical NOX subunit p22^phox^ with small interfering RNA, indicating that increased basal level of superoxide impairs RPT function [137]. Furthermore, in Sprague-Dawley rats, oxidative stress impairs Ang II-mediated regulation of NHE3 [138].

## 6. Oxidative Stress and Regulation of Na/K-ATPase

Many stimuli including hypoxia and dopamine induce a cell- and tissue-specific endocytosis and exocytosis of Na/K-ATPase and a change in Na/K-ATPase activity [33, 61, 139]. Ouabain-stimulated ROS generation functions as a second messenger in ouabain-activated Na/K-ATPase signaling in isolated rat cardiac myocytes [82, 140–142]. Binding of ouabain to the Na/K-ATPase activates Src kinase, which in turn transactivates EGFR, leading to activation of the Ras-Raf-MEK-ERK pathway [32, 39, 63]. Ras activation leads to the activation of MAPK and increase in [Ca^2+^]_i_ which result in opening of mitochondrial ATP-sensitive K^+^ channels [142] and generation of mitochondrial ROS [82, 141]. ROS subsequently activate NF-*κ*B [141, 143] and slow [Ca^2+^]_i_ oscillations at nanomolar ouabain concentrations [40]. Additionally, ouabain-induced generation of ROS in neonatal myocytes is antagonized by overexpression of a dominant negative Ras as well as myxothiazol/diphenyleneiodonium, indicating a mitochondrial origin of the Ras-dependent ROS generation [82]. Ouabain also stimulates ROS generation in other cell types [144–146]. Conversely, oxidative stress can activate the Na/K-ATPase signaling. Both a bolus of H_2_O_2_ and exogenously added glucose oxidase (which generates a sustained low level of H_2_O_2_ by consuming glucose) activates Na/K-ATPase signaling in cardiac myocytes [140]. Pretreatment with the antioxidant N-acetyl cysteine (NAC) prevents ouabain-Na/K-ATPase signaling and its downstream effects. Moreover, infusion of CTS causes ROS generation and protein oxidation in experimental animals [80, 147].

 The redox sensitivity of the Na/K-ATPase was first demonstrated in electric eels with treatment of H_2_O_2_ [148]. This phenomenon was further observed in a wide range of animal species, tissues, and other species of ROS such as hypochlorous anion, hydroxyl radicals, superoxide, hyperchlorite anions, and singlet oxygen. It has been shown that the Na/K-ATPase in skeletal muscle is redox-sensitive and infusion of NAC attenuated ROS-mediated inhibition of maximal pump activity [149]. In dog kidney, oxidative modification of kidney Na/K-ATPase by H_2_O_2_ was accompanied by a decrease in the amount of sulfhydryl (SH) groups [150] and, importantly, oxidative modification can result in formation of Na/K-ATPase oligomeric structure [151]. The differences in the number, location, and accessibility of SH groups in Na/K-ATPase isozymes might predict their oxidative stability [152]. Different antioxidant enzymes, natural or synthetic antioxidants, and some inhibitors of oxidase activity can attenuate ROS mediated inhibition of Na/K-ATPase activity. ROS are known to inhibit Na/K-ATPase activity in different types of cells as well. Interestingly, the Na/K-ATPase in rat cerebellar granule cells are redox-sensitive with an “optimal redox potential range,” where ROS levels out of this “optimal range” are capable of inhibiting Na/K-ATPase activity [153]. While the Na/K-ATPase does not contain heme groups, it does contain cysteine residues located in *α* subunit cytosolic loops which may determine the redox sensitivity of the *α* subunit. The sensitivity of the Na/K-ATPase to redox and oxygen status, the regulatory factors which govern these interactions, and the implied molecular mechanism were recently reviewed [154].

Increases in ROS can oxidize the Na/K-ATPase ***α*/***β***** subunits and its independent regulator FXYD proteins. This oxidation inhibits its activity and promotes its susceptibility to degradation by proteasomal and endosomal/lysosomal proteolytic pathways in different cell types including cardiac myocytes, vascular smooth muscle cells, and RPTs [155–166]. It appears that the oxidized modification of the Na/K-ATPase is a reversible, redox-sensitive modification. However, purified enzyme has also been shown to be irreversibly inhibited upon exposure to hydrogen peroxide, the superoxide anion, and the hydroxyl radical [158]. The regulation of renal Na/K-ATPase ***α*** 1 by oxidants is not dependent on the ouabain sensitivity of the ***α*** 1 subunit *per se*. It appears that the ***α*** 2 and ***α*** 3 subunits are more sensitive to oxidants than the ***α*** 1 subunit, and ouabain-sensitive ***α*** 1 (canine) and insensitive-***α*** 1 (rat) have similar sensitivity to oxidants, suggesting the regulation of ***α*** 1 by ROS is not species specific. Furthermore, ROS accelerates degradation of the oxidatively damaged Na/K-ATPase and the ***α*** 2 and ***α*** 3 subunits, which also appear to be more susceptible to degradation than the ***α*** 1 subunit [159]. These studies indicate that differential oxidant sensitivities of the Na/K-ATPase subunits are dictated by the primary sequences of different subunits and different subunit compositions of the various tissues may contribute to their relative susceptibilities to oxidant stress. In the RPT cell line originated from WKY rats, cadmium, a ROS generator, stimulates ROS production and causes a toxic oxidative damage of the Na/K-ATPase. Oxidative damage increases Na/K-ATPase degradation through both the proteasomal and endo-/lysosomal proteolytic pathways [163]. In purified renal Na/K-ATPase, peroxynitrite (ONOO^−^) causes tyrosine nitration and cysteine thiol group modification of the Na/K-ATPase, but only cysteine thiol group modification is implied in the inhibition of the enzyme activity since glutathione is unable to reverse the inhibition [167]. In isolated rat renal RPTs, peroxynitrite and its signaling participates in Ang II-induced regulation of renal Na/K-ATPase activity [168].

Ang II inhibits the Na/K-ATPase via PKC-dependent NOX activation. The dependence of Ang II-induced Na/K-ATPase inhibition on NOX and superoxide, as well as reversible oxidative modification of the Na/K-ATPase, strongly suggests a role for redox signaling [164–166]. In cardiac myocytes and pig kidney, oxidative stress induces glutathionylation of the *β*1 subunit of the Na/K-ATPase. In purified pig renal Na/K-ATPase, peroxynitrite inhibits the enzymatic activity by stabilization of the enzyme in an E_2_-prone conformation. At the same time, FXYD proteins reverse oxidative stress-induced inhibition of the Na/K-ATPase by facilitating deglutathionylation of the *β*1 subunit. Moreover, both tyrosine kinase c-Src and cell membrane structural component lipid rafts, which are critical in Na/K-ATPase/c-Src signaling, are also critical in redox signaling platform formation [169–172]. These observations, along with the fact that c-Src is redox-sensitive [173] and its activation regulates NOX-derived superoxide generation [174], suggests a redox-sensitive Na/K-ATPase/c-Src signaling cascade and its possible role in ROS regulation, although the mechanism is not clear. Our unpublished data suggest that certain basal levels of ROS might be required for the initiation of ouabain-Na/K-ATPase/c-Src signaling. In LLC-PK1 cells, pretreatment with higher concentrations of NAC (5 and 10 mM for 30 min), but not with lower concentration of 1 mM, can prevent ouabain-stimulated c-Src activation and the redistribution of Na/K-ATPase and NHE3. This suggests that ROS may stabilize Na/K-ATPase in a certain conformational status in order to facilitate ouabain binding to the Na/K-ATPase ***α*** 1 subunit and favor ouabain-Na/K-ATPase/c-Src signaling. Some pertinent questions remain to be resolved, such as how ROS interacts with and influences the Na/K-ATPase/c-Src signaling cascade and whether ouabain-induced ROS boosts the Na/K-ATPase signaling by a positive feedback mechanism and chronically desensitizes the signaling cascade by stimulating Na/K-ATPase/c-Src endocytosis (Figure 2).

## 7. Oxidative Stress and Regulation of Renal Function

ROS function as important intracellular and extracellular second messengers to modulate many signaling molecules. Physiological concentrations of ROS play an important role in normal redox signaling, while pathological levels of ROS contribute to renal and vascular dysfunction and remodeling through oxidative damage [175–177]. Genetic factor(s) partially contribute to high basal ROS levels and the development of hypertension [178, 179].

 Oxidative stress has been shown to regulate BP and sodium handling in various animal models. High salt intake increases oxidative stress, and this has important implications for the regulation of cardiovascular and renal systems. An increase in oxidative stress is both a cause and consequence of hypertension [177, 180–183]. Renal NOX is present in the renal cortex, medulla, and vasculature. NOX, the major source of superoxide in the kidney, is of particular interest because of its prominent expression and implication in pathophysiology. In the kidney, increased oxidative stress influences a number of physiologic processes including renal sodium handling in the proximal tubule [137, 168, 184, 185] and thick ascending limb [186], renal medulla blood flow [183, 187–190], descending vasa recta contraction [191, 192] in addition to interactions with other regulatory systems such as the dopamine signaling pathway [193]. Increased oxidative stress has been shown to contribute to salt sensitivity [194, 195]. In macula densa cells, NOX isoform Nox2 is responsible for salt-induced superoxide generation, while Nox4 regulates basal ROS [196]. In the medullar thick ascending limb of the loop of Henle, both exogenous and endogenous superoxides stimulate sodium absorption [197]. Also, in this nephron segment, NOX-induced generation of superoxide enhances sodium absorption by reduction of the bioavailability of nitric oxide (NO) to prevent NO-induced reduction of NaCl absorption [198], which contributes to salt-sensitive hypertension observed in Dahl salt-sensitive rats [190].

In PRTs, in particular, increases in ROS inhibit the Na/K-ATPase as well as the apical NHE3 and sodium/glucose cotransporter, in order to promote RPT sodium excretion under certain circumstances [137, 168, 184, 185]. While elevated basal level of superoxide inhibits proximal tubule NHE3 activity and fluid reabsorption in SHR rats in comparison to WKY rats [137], peroxynitrite and its signaling participates in Ang II-induced regulation of renal Na/K-ATPase activity in isolated rat renal RPTs [168]. In the pathogenesis of diabetic nephropathy, a high level of glucose-induced ROS generation induced by stimulation of mitochondrial metabolism and NOX activity in RPT primary cultures leads to inhibition of the expression and activity of the sodium/glucose cotransport system [185]. In male Wistar rats, a high salt diet (3% NaCl for 2 weeks) promotes sodium/water excretion and urinary 8-isoprostane excretion, a marker of oxidative stress, which can be attenuated by treatment with apocynin, an NOX inhibitor. The salt loading leads to increased generation of ROS and a state of oxidative stress in the cortex but not to such a degree in the medulla [199].

RPT sodium and/or fluid absorption in the normal rat is reduced by inhibition of NO synthesis, while NO promotes RPT Na^+^ and/or fluid reabsorption [200]. In immortalized and freshly isolated RPTs from the WKY and SHR rats, the basal level of membrane NOX activity is greater in SHRs [201]. Moreover, NOX-induced superoxide generation inhibits RPT fluid reabsorption in SHRs [137]. In Sprague-Dawley rats treated with the oxidant L-buthionine sulfoximine, Ang II overstimulates RPT Na/K-ATPase and NOX and leads to increased sodium reabsorption, which is prevented by administration of the superoxide scavenger Tempol [202].

High salt diet, which is well documented for its stimulation of systematic oxidative stress, regulates the activity and distribution of the Na/K-ATPase and NHE3 in different animal models [203–207]. PRT sodium reabsorption significantly affects water and sodium homeostasis by regulating redistribution of ion transporters in response to high salt intake [208]. Regulation of NHE3 activity and distribution as well as PRT fluid reabsorption contribute to the development and maintenance of hypertension in young and adult SHR rats [127, 137, 209].

It has become clear that antioxidant agents such as Tempol and enzymes such as heme oxygenase-1 (HO-1) exhibit a beneficial and protective effect on BP in various animal models of hypertension. As an example, inhibition of HO activity reduces renal medullary blood flow [84, 210, 211], total renal blood flow (RBF) [212], glomerular filtration rate (GFR), and renal production of nitric oxide [213]. Inhibition of HO-1 increases mean arterial pressure in Sprague-Dawley rats [214, 215] and SHR rats [216]. Induction of HO-1 reduces BP in SHR rats [217–220]. The effect of HO-1 on BP is presumably through the carbon monoxide (CO)/HO system and depression of cytochrome p-450-derived 20-HETE. In the Dahl salt-dependent model of systemic hypertension, induction of HO-1 occurred in the vasculature and is accompanied by endothelial dysfunction [221, 222]. In Dahl salt-sensitive rats, a high salt diet increases HO-1 expression and CO generation in aorta and coronary arteries [221, 223], and, in Dahl salt-sensitive rats fed low salt diet, induction of HO-1 expression attenuates oxidative stress and reduces proteinuria and renal injury [224]. However, most studies examining the contribution of HO-1 to BP regulation have focused on the vasculature, that is, pressure-natriuresis and arterial BP, and so the role of HO-1 in RPT-mediated sodium handling is still poorly understood. Nevertheless, increased HO expression in RPTs could result in an increased ability to buffer locally produced oxidants, leading to their neutralization.

The beneficial effect of antioxidants is controversial and not seen in most clinic trials with administration of antioxidants (reviewed in [177, 225]). The Dietary Approaches to Stop Hypertension (DASH) study and subsequent studies have demonstrated that lower BP associated with reduced dietary salt intake may be related to reductions in oxidative stress [6, 226–228]. Interestingly, however, while a combination antioxidant supplement (with an ascorbic acid, synthetic vitamin E, and *β*-carotene) had no improvement on BP after 5-year treatment [229], another combination antioxidant supplement (zinc, ascorbic acid, *α*-tocopherol, and *β*-carotene) did result in a significant reduction in systolic BP [230]. Other studies have also shown that certain antioxidants, such as glutathione and vitamin C, have a blood-pressure-lowering effect [231, 232]. However, antioxidant supplementation may be ineffective or even dangerous [233] due to the possible “over-antioxidant-buffering” effect of excessive antioxidant supplementation. In this scenario, excess antioxidants might become pro-oxidants (by providing H^+^) if they cannot promptly be reduced by the following antioxidant in the biological antioxidant chain. Thus, it appears that the balance of the ROS status, within a physiological range, may be more important to maintain beneficial ROS signaling.

## 8. Perspective

Renal sodium handling is a key determinant of blood pressure. ROS status, among others, is an important regulator of vasculature and sodium handling. However, the effect of ROS and Na/K-ATPase, especially of their interaction, on RPT sodium reabsorption has only been explored in a limited fashion. Coordinated regulation of two major ion transporters, the basolateral Na/K-ATPase and the apical NHE3, has been implicated in the counterbalancing of high salt intake (volume expansion) mediated blood pressure increase. The Na/K-ATPase/c-Src signaling regulates this coordinated regulatory mechanism and impairment of this signaling cascade contributes to experimental Dahl salt-sensitive hypertension. Both the Na/K-ATPase (*α* and *β* subunit) and its proximal signaling partner c-Src are redox sensitive, suggesting a redox-sensitive Na/K-ATPase/c-Src signaling complex. However, the mechanisms remain largely to be elucidated since the available data is limited [184, 234]. Nevertheless, some pertinent questions remain to be addressed. In the future, it will be important to explore whether ROS signaling is a link between the Na/K-ATPase/c-Src cascade and NHE3 regulation and how oxidative stress stimulated by high salt and CTS regulates Na/K-ATPase/c-Src signaling in renal sodium handling and fibrosis.
